# Proximal endoscopic repair of the hamstring tendons: a cadaveric anatomical study of posterior hip portals

**DOI:** 10.1093/jhps/hnad001

**Published:** 2023-03-22

**Authors:** Lucas Verissimo Ranzoni, Matheus Almeida Guberovich, Leandro Ejnisman, Helder Souza Miyahara, Ehud Rath, Henrique Melo de Campos Gurgel, Alfredo Luiz Jacomo

**Affiliations:** Department of Pathophysiology, Faculty of Medicine, USP, Av. Dr. Arnaldo, 455 - Cerqueira César, São Paulo, SP 01246-903, Brazil; Department of Pathophysiology, Faculty of Medicine, USP, Av. Dr. Arnaldo, 455 - Cerqueira César, São Paulo, SP 01246-903, Brazil; Orthopedics Department, Hospital Israelita Albert Einstein, Dr. Ovídio Pires de Campos Street, 333, Cerqueira Cesar, São Paulo, SP 05403-010, Brazil; Orthopedics Department, Hospital das Clínicas—FMUSP, Dr. Ovídio Pires de Campos Street, 333, Cerqueira Cesar, São Paulo, SP 05403-010 Brazil; Orthopedics Department, Tel Aviv University School of Medicine, Tel Aviv-Yafo 6997801, Israel; Orthopedics Department, Hospital das Clínicas—FMUSP, Dr. Ovídio Pires de Campos Street, 333, Cerqueira Cesar, São Paulo, SP 05403-010 Brazil; Department of Surgery, Faculty of Medicine, USP, Av. Dr. Arnaldo, 455 - Cerqueira César, São Paulo, SP 01246-903, Brazil

## Abstract

Arthroscopy and endoscopic hip surgery have attracted increasing attention in the orthopedic field. In the case of arthroscopy, portals and their relationships with neurovascular bundle structures at risk are well established. However, studies on endoscopic portals used for the repair of hamstring tendon injuries are insufficient. Hamstring injuries are the most common muscle injury in sports medicine, and up to 12% can present as a tendon rupture. Endoscopic surgery is advantageous because it has a lower rate of bleeding and avoids excessive handling of the gluteal muscles. The objective of this study is to perform an anatomical evaluation of endoscopic portals for hamstring repair and measure their distance to neurovascular structures—mainly sciatic nerve and posterior femoral cutaneous nerve (PFCN). Fifteen hips from frozen and formalized cadavers were evaluated. Specimens that showed any modification in their anatomy were excluded. Portals were simulated using Steinmann pins, and anatomical dissection was performed. Distances from neurovascular structures were measured using a digital caliper. Four male cadaver hips (26%) and eleven female cadaver hips (74%) were included. Two dissected hips presented PFCN injury through the posterolateral portal— mean 20.28 mm (±8.14), and one through the distal accessory portal— 21.87 mm (±12.03). The injury rate for PFCN was 3/15 or 20%. None of the portals presented sciatic nerve injury. Conclusion: There is an imminent risk of nerve injury to the PFCN by performing the lateral portals for hamstring repair. To avoid this, we recommend starting the procedure through the most medial (posteromedial) portal, and the other portals must be performed under direct visualization.

## INTRODUCTION

Arthroscopy and endoscopic hip surgery are widespread procedures in the orthopedic field, especially in the last two decades, with different indications for a minimally invasive approach to articular and non-articular structures. The first arthroscopic hip surgery to treat acetabular labrum injury dates back to 1986; and with the development of technology and surgical technique, especially after the 2000s, the procedure has become widely used [1, 2].

The surgical technique for hip arthroscopy involves a series of distinctive steps. The patient should be positioned on an orthopedic table, limb traction is used to increase the joint space and the portals are established using an image intensifier. There is specific instrumentation for hip arthroscopy, given the thick muscular layer and subcutaneous cellular tissue around the joint [1–3].

Several studies have shown the anatomical relationships of the portals of hip arthroscopy with adjacent neurovascular structures [4, 5]. However, the literature lack anatomical studies on the portals used in the treatment of injuries of the origin of the hamstring muscles (semimembranosus, semitendinosus and biceps femoris muscles).

The hamstrings are responsible for hip extension, knee flexion and limb external rotation, perform hip propulsion, stabilization and deceleration; and along with the gluteal musculature are used in gait. They also work as pelvic decelerators, together with the rectus abdominis muscle. Their insertion is divided into a conjoined tendon of the long head of the biceps femoris and semitendinosus inserted into the ischium in a crescent shape and the semimembranosus tendon with its insertion just medial and anterior to this conjoint tendon. Except for the short head of the biceps femoris, a monoarticular muscle innervated by the common fibular nerve, the rest of the musculature is biarticular and innervated by the tibial nerve [4–6].

Hamstring injuries or ruptures are the most common in sports medicine—up to 30%—and are responsible for the longest time away from the sport [6]. They usually result from eccentric loading, with the knee extended and the hip flexed. Although studies show a relationship of hamstring injuries with contact sports, 90% of them happen in the absence of contact [7].

Tendon ruptures correspond to ∼12% of these injuries, and a complete anamnesis and a good knowledge of the physical examination are essential for the early diagnosis and adequate management, aiming at good functional results. Surgical treatment for ruptures presents good outcomes [8, 9].

The repair of the injuries can be open or endoscopic. The advantages of endoscopic access are minimally invasive, less blood loss, no need for retraction of the gluteus maximus muscle and close evaluation of the tendon, even for partial injuries. On the other hand, the disadvantage is mainly the possibility of damage to neurovascular structures caused by establishing the portals [10–12].

During the surgical procedure, the patient is positioned prone, and a number of portals are described in the literature [13–16].

**Purpose:** Considering the lack of literature, our proposal is to evaluate the safety of endoscopic portals for the repair of the hamstring tendons, verifying the distance between these accesses and important structures, such as the sciatic nerve and posterior femoral cutaneous nerve (PFCN), to determine the correct and safe measurements for this surgical technique. **Hypothesis:** Identify an imminent risk of injury to the PFCN and sciatic nerve in endoscopic approaches for hamstring repair.

## METHODS

This is a cross-sectional and descriptive anatomical study, which was approved by the local ethics committee. Fifteen formolized cadaveric hips were selected. Adult cadavers (over 18 years old), of both sexes, without compromising their original anatomy, either by previous surgeries, scars, retractions or any variation, were included in the study. All selected cadavers had the site to be studied dissected for the first time.

**Fig. 1.**
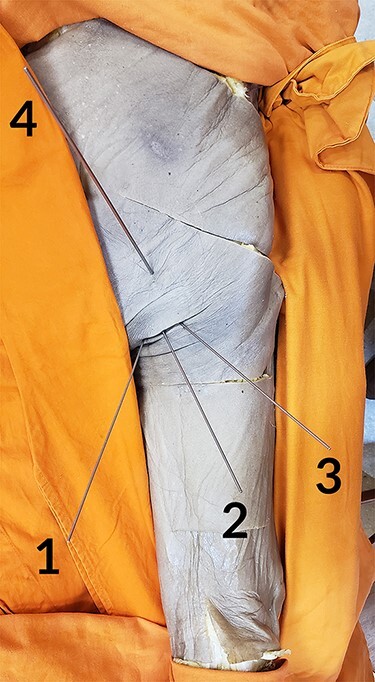
Simulated portals: PM (1), DA (2), PL (3) and PA (4).

**Fig. 2.**
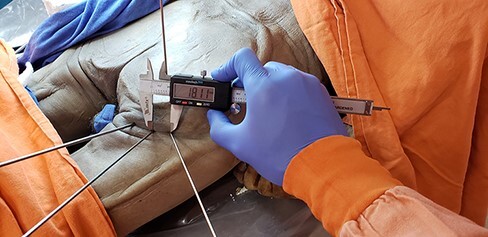
Example of the measurement of interportal distance.

There are a variety of portals described in the literature [3, 10, 11, 13–16]. We considered four portals for the study, according to a previous study [3]. This choice was made because there were a greater number of portals to analyze and discuss. In addition, those are the accesses that our team is familiar with. We start with a primary distal accessory (DA) portal, 1 cm lateral to the ischial tuberosity and close to the gluteal fold; a posteromedial (PM) portal, 2–4 cm medial to the DA; a posterolateral (PL) portal, 2–4 cm lateral to the DA; and a proximal accessory (PA) portal, 5–7 cm proximal to the DA (see Fig. 1). They were simulated using Steinmann wires, with subsequent anatomical dissection of the hips in order to identify adjacent neurovascular structures at risk, specifically in this topography the sciatic nerve and the PFCN [17]. The distance between these structures and the portals was measured in millimeters using a digital caliper (Fig. 2).

Data are presented as mean (±standard deviation) for normally distributed variables, median (interquartile range) for other continuous variables and frequencies (valid %) for categorical variables. All analyses were performed using R (R Foundation for Statistical Computing, Vienna, Austria, 2018).

## RESULTS

Demographic analysis of the cadavers showed a median age of 66 years (8) years and a mean height of 1.68 (±0.05), according to data collected from the identification tags. Laterality: 7 right hips (46.6%) and 8 left hips (53.3%). Regarding sex, 4 male cadaver hips (26%) and 11 female cadaver hips (74%) were included.

**Fig. 3.**
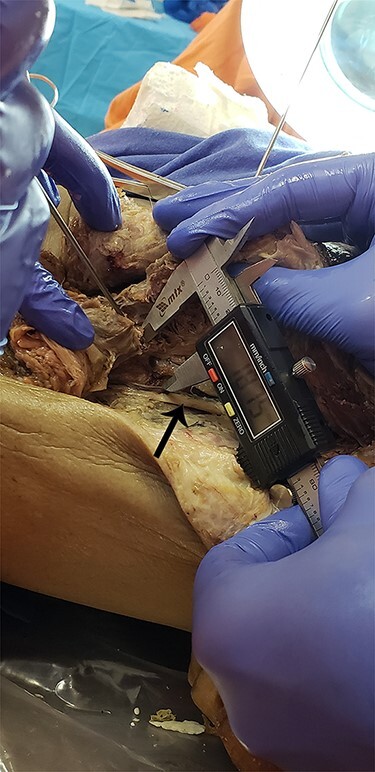
Example of the measurement from portals to target structures. Arrow: sciatic nerve.

**Graph 1.**
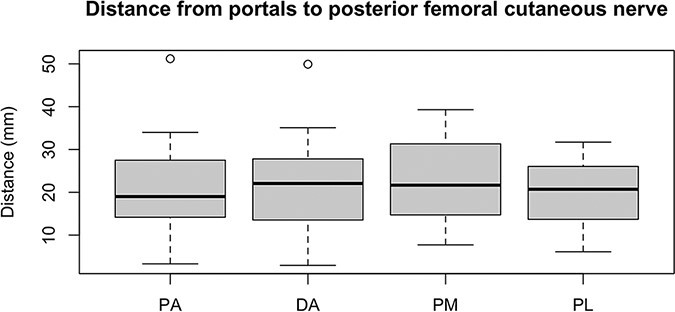
Distance from portals, in millimeters, to the PFCN.

**Graph 2.**
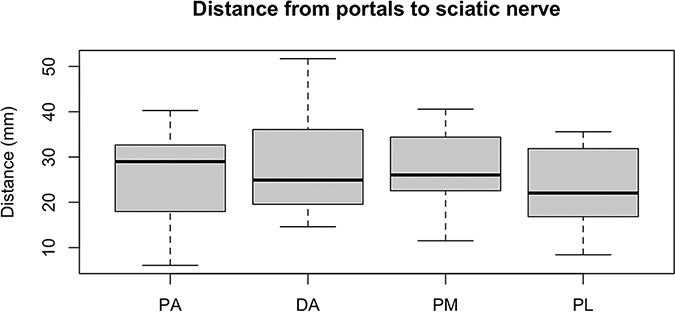
Distance from portals, in millimeters, to the sciatic nerve.

**Graph 3.**
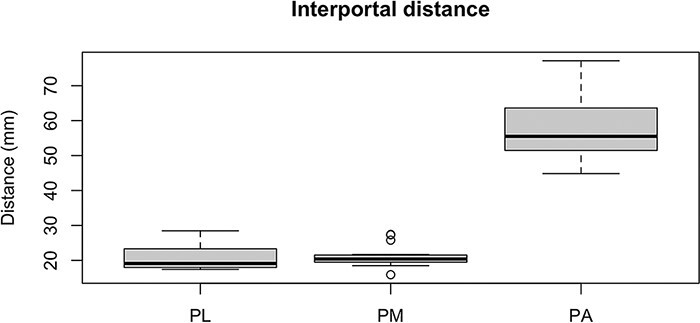
Interportal distance, in millimeters, taking the distal accessory portal as reference.

The measurements collected were the distance between portals, taking the distal accessory portal as reference, and the distances from the Steinmann wires to the sciatic nerve and PFCN. Figs. 2 and 3 show examples of the measurements. With the measurements collected, we verified the results shown in Table I and the Graphs 1–3, in millimeters.

**Table I.** Distances in millimeters from portals to study structures—sciatic nerve and PFCN

DA and PL distance, median (interquartile range)	19.12 (IQR = 5.34)
DA and PM distance, median (interquartile range)	20.39 (IQR = 2.04)
DA and PA distance, mean (±SD)	57.31 (±9.19)
PL and sciatic nerve distance, mean (±SD)	23.49 (±8.47)
PL and PFCN distance, mean (±SD)	20.28 (± 8.14)
DA and sciatic nerve distance, mean (±SD)	27.48 (± 10.26)
DA and PFCN distance, mean (±SD)	21.87 (± 12.03)
PM and sciatic nerve distance, mean (±SD)	27.45 (± 8.53)
PM and PFCN distance, mean (±SD)	23.14 (± 10.04)
PA and sciatic nerve distance, mean (±SD)	25.40 (± 10.29)
PA and PFCN distance, mean (±SD)	20.536 (± 12.45)

One dissected hip was excluded because the specimen had been submitted to a hip arthroplasty. In two hips, direct injury to the PFCN was observed in the course of the Steinmann wire through the PL portal—20.28 (±8.14). In one of them, direct injury to the PFCN was also observed in the DA access—21.87 (±12.03). The PFCN injury rate in this study, therefore, was 3/15 (20%). None of the dissected hips showed direct injury to the sciatic nerve in the portal simulation.

## DISCUSSION

In this cadaveric study, the local anatomy of the posterior hip was described in relation to the arthroscopic hamstring repair. We found that the structures at risk are especially the sciatic nerve and the PFCN. Other structures such as the inferior gluteal bundle and the pudendal nerve were at a safe distance from the portals, as verified by Su *et al*. [17]. We considered the cluneal nerves as branches of the PFCN, and therefore in this study, only this main sensory branch was evaluated.

We verified direct injury caused by Steinmann wires in two cadavers, one of them for both the DA and PL portals and the other only for the PL. In this case, we considered an overall risk of injury to the PFCN of 6.67% (3/45) and to the PL access of 13.34% (2/15). Thus, we found a greater risk of injury to the structures - mainly to PFCN - when establishing the lateral portal. Although there are various guidelines regarding the portals and which one should be the first to be established during surgery, we strongly consider the first portal to be always as medial as possible, in this case the PM, precisely to avoid damage to the sciatic nerve and PFCN.

Another relevant aspect that we considered was the position of the limbs. All specimens were formolized and frozen in the supine position, the vast majority (12/15; 80%) had the hip in external rotation and it was not possible to manipulate the joint to a neutral position. This can lead to an approximation of the structures with the paths of the portals [16, 18]. Laskovski *et al*. even guide a safer position, i.e. lateralizing the sciatic nerve with abduction of 45° of the hip to be operated and in neutral rotation.

### Limitations

In addition to the situation of joint stiffness that we found in the frozen and formolized cadavers, we also had limitations regarding the cutaneous anatomy of the specimens, which were frozen in the horizontal dorsal decubitus position. This leads to losing the skinfolds, especially the gluteal crease. The thick cutaneous layer also made the palpation of the ischial tuberosity difficult. However, although this difficulty existed initially, in all cadavers it was possible to perform the assessment safely, reaching the anatomical target, which was confirmed after dissection.

We performed all measurements with the cadavers in the ventral position, as we found it to be the most used position in the literature. Other positions such as lateral or supine are also valid, but they were not addressed in this study. Another limitation is the lack of a second measurement in order to calculate inter and intra-rater reliability measures. However, during the dissections we considered a second measurement would not add to our research. Moreover, previous studies on the anatomy of portals also described only one measurement [4, 19–21].

Despite these limitations, this study has great significance for this surgical technique, which is still not widespread, especially due to the large learning curve, demonstrating its safety and benefit for patients with hamstring injuries. Further studies may provide more information, especially regarding patient-positioning concerns.

## CONCLUSION

In conclusion, we found that there is greater safety in the creation of portals starting as medially as possible, with the PM access, which is further away from the structures at risk. There is a risk of injury to the PFCN when performing the lateral portals with no direct visualization. Therefore, these portals must be performed under direct visualization, for greater safety, to avoid iatrogenic injuries.

## Data Availability

All statistical data are available for submission, as needed, by contacting the corresponding author.
